# Phylogenomic Analysis and Functional Characterization of the APETALA2/Ethylene-Responsive Factor Transcription Factor Across Solanaceae

**DOI:** 10.3390/ijms252011247

**Published:** 2024-10-19

**Authors:** Fan Yang, Songxue Han, Yangxin Zhang, Xiangxiang Chen, Wenxian Gai, Tao Zhao

**Affiliations:** College of Horticulture, Northwest A&F University, Yangling 712100, China; xnyangfan@nwafu.edu.cn (F.Y.); 15100277939@163.com (S.H.); yangxin.zhang@nwafu.edu.cn (Y.Z.); xxchen@nwafu.edu.cn (X.C.)

**Keywords:** Solanaceae, AP2/ERF, phylogenomics, synteny, gene duplications, flowering time

## Abstract

The AP2/ERF family constitutes one of the largest groups of transcription factors in the Solanaceae. AP2/ERF contributes to various plant biological processes, including growth, development, and responses to various stresses. The origins and functional diversification of AP2/ERF within the Solanaceae family remain poorly understood, primarily because of the complex interactions between whole-genome duplications (WGDs) and tandem duplications. In this study, a total of 1282 AP2/ERF proteins are identified from 7 Solanaceae genomes. The amplification of AP2/ERF genes was driven not only by WGDs but also by the presence of clusters of tandem duplicated genes. The conservation of synteny across different chromosomes provides compelling evidence for the impact of the WGD event on the distribution pattern of AP2/ERF genes. Distinct expression patterns suggest that the multiple copies of AP2/ERF genes evolved in different functional directions, catalyzing the diversification of roles among the duplicated genes, which was of great significance for the adaptability of Solanaceae. Gene silencing and overexpression assays suggest that ERF-1 members’ role in regulating the timing of floral initiation in *C. annuum*. Our findings provide insights into the genomic origins, duplication events, and function divergence of the Solanaceae AP2/ERF.

## 1. Introduction

AP2 (APETALA2)/ERF (Ethylene-responsive factor) protein, comprising of the conserved AP2/ERF DNA-binding domain, is an important transcriptional factor and can regulate the expression of a set of downstream, endogenous, and abiotic stress-inducible genes by recognizing and binding to a cis-acting regulatory element as the binding site in their promoter region [1,2,3]. The AP2/EFR gene is primarily known for its AP2 domain, a highly conserved DNA-binding domain consisting of approximately 60–70 amino acids [4,5]. This domain contains two β-sheets and an α-helix, forming a three-dimensional structure that recognizes specific DNA sequences. The AP2 domain is characterized by a YRG (tyrosine–arginine–glycine) motif and a WLG (tryptophan–leucine–glycine) motif, which are essential for DNA binding [6]. These motifs allow the AP2 proteins to bind to GCC-box or DRE/CRT (dehydration-responsive element/C-repeat) elements in the promoters of target genes [1,2,7,8]. This conserved sequence (CCGAC), named the C-repeat/dehydration response element (CRT/DRE), is present in the promoters of functional target genes [1,2,7,8]. AP2/ERF genes have been shown to play critical roles not only in plant response to various stresses but also in plant growth and development [3].

The involvement of AP2/ERF has been observed in the response to diverse stress conditions. Low temperature is an adverse environmental factor commonly encountered in plants’ native habitats, affecting their growth and development. Up to now, a large number of AP2/ERF genes have been reported to show a variety of roles in freezing tolerance. Transcriptome analysis in Arabidopsis indicated that 12% of the cold-regulated genes were confirmed members of the CBF cold response pathway [9]. Overexpression of Arabidopsis AP2/ERF genes *CBF1* or *CBF3* enhanced cold tolerance in transgenic Arabidopsis [2,10]. A major quantitative trait locus of freezing tolerance, which mapped to a region of *Medicago truncatula* chromosome 6, accounted for 40% of the phenotypic variation [11], and a tandem array of 11 *CBF*/*DREB1* genes were isolated from this region and revealed high polymorphism between the freezing-tolerant and freezing-sensitive accession [12]. The soybean AP2/ERF gene *GmDREB1* enhances drought tolerance in transgenic soybeans by interacting with GmERFs [13]. In contrast, the rice AP2/ERF gene *OsERF71* mediates a drought resistance pathway by recruiting factors involved in cell wall modification [14]. Additionally, overexpressing rice *OsERF19* increases plant tolerance to salt stress [15]. Under heat stress, the Arabidopsis ERF1 positively regulates heat tolerance by activating the expression of heat-responsive genes (*HSFA3* and *HSPs*) [16]. AP2/ERFs are also involved in other stress responses, such as heavy metals [17], osmotic [18], and nutritional element stress [19].

The role of AP2/ERF transcription factors in responding to environmental stress is just one aspect of their numerous biological functions; they also play a crucial role in plant growth and development. A subset of AP2/ERF transcription factors, including Abscisic Acid Repressor1 and ERF109, is rapidly induced by wounding and acts as a signal to initiate auxin biosynthesis [20]. Rice ERF3-regulated *RR2* (type-A RR gene) expression plays a role in the initiation of crown roots, whereas the interaction between *ERF3* and *WOX11* (WUSCHEL-related homeobox gene) likely suppresses *RR2* during crown root elongation [21]. Furthermore, AP2/ERF genes act as flowering repressors. Several Arabidopsis AP2-like genes (TOE1, TOE2, TOE3, SMZ, SNZ) negatively regulate the induction of flowering [22,23]. A pepper CaAP2 transcription factor has been proven to be a candidate gene for flowering repression and for controlling natural variation in flowering time [24]. Similar gene functions have also been identified in maize [25] and barley [26]. AP2/ERF transcription factors are also involved in the regulation of plant metabolism. Members of the citrus AP2/ERF family regulate flavonoid synthesis by controlling type IV chalcone isomerase [27]. Additionally, *CitERF71* plays a pivotal role in the transcriptional regulation of *CitTPS16* by directly interacting with its promoter, thereby influencing E-geraniol production in citrus fruits [28]. In apples, the ERF transcription factor MdERF38 enhances anthocyanin biosynthesis in response to drought stress [29]. Conversely, in red-skinned pears, ERF4.1 and ERF4.2 repress anthocyanin biosynthesis [4].

The diversity and complexity of AP2/ERF gene functions arise from the large number of family members. The AP2/ERF family has been identified and analyzed in many plant species, such as Arabidopsis (*Arabidopsis thaliana*) [30], rice (*Oryza sativa*) [30], grape (*Vitis vinifera*) [31], soybean (*Glycine max*) [32], poplar (*Populus tricocarpa*) [33], peach (*Prunus persica*) [34], and pepper (*Solanum tuberosum*) [35]. And 145 Arabidopsis AP2/ERF were classified into five categories based on AP2 domain similarity, namely, AP2, RAV, SOLOIST, DREB, and ERF [36]. The AP2 subfamily comprises proteins with two highly similar and serially repeated AP2 domains. The RAV-like AP2/ERF transcription factor contains one AP2 domain and one B3 domain. The AP2 gene grouping of many other plant species is still based on the AP2 gene of Arabidopsis [30,33,34,35]. Significant progress has been made in understanding the characteristics of AP2/ERF genes in individual species and the role of development and stress regulation. The Solanaceae is a large group of flowering plants belonging to the dicotyledon class, comprising over 90 genera and more than 2500 species, which are widely distributed globally, particularly abundant in tropical and temperate regions [37]. Solanaceae family, the third most significant plant taxon, is economically significant, including several food crops such as tomato and potato, as well as various medicinal and ornamental plants [5,38]. However, research focused on elucidating the systematic evolution of AP2/ERF genes across the Solanaceae family remains notably lacking. This highlights the need for more extensive studies to gain deeper insights into the evolutionary history and functional implications of AP2/ERF across this diverse Solanaceae family.

This study aims to elucidate the systematic evolution of AP2/ERF genes, a gene family that plays crucial roles in plant development, stress responses, and hormonal signaling. The AP2/ERF family was selected for this study due to its extensive involvement in various aspects of plant biology. Furthermore, the AP2/ERF family is one of the largest and most diverse transcription factor families in plants, providing a rich source of information for understanding the evolutionary dynamics of gene families. By conducting comprehensive phylogenetic analyses, we seek to uncover the evolutionary relationships and diversification patterns within this gene family across multiple Solanaceae species. By combining phylogenetic reconstruction and synteny analysis, this study traced the ancestral origin of AP2/ERF in Solanaceae, followed by investigating the broad expression profiles in *Solanum lycopersicum* and *Capsicum annuum*. Further functional analysis indicates that members of the ERF-1 subfamily of AP2/ERF play a significant role in the transition from vegetative to reproductive growth. Through these efforts, we aim to provide a deeper understanding of the evolutionary mechanisms that have shaped the AP2/ERF gene family and their contributions to plant development. Ultimately, our findings will not only enhance our knowledge of the genetic basis of key Solanaceae traits but also offer valuable insights for future breeding and biotechnological applications.

## 2. Results

### 2.1. Phylogenetic Reconstruction of AP2/ERF Proteins and Their Distribution in Solanaceae

**Figure 1:**
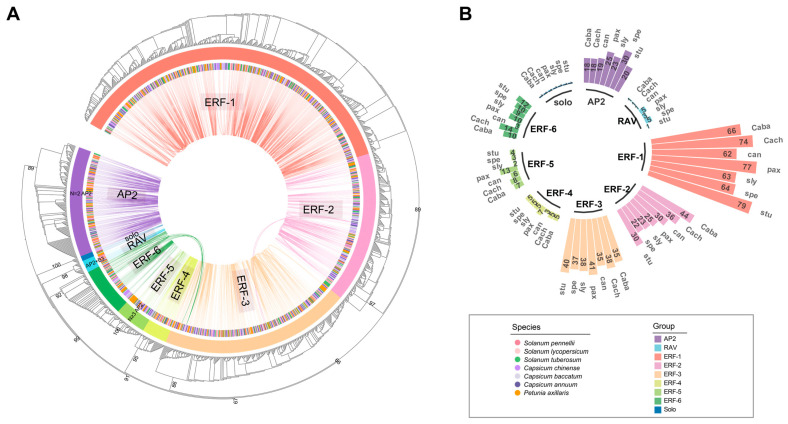
Phylogenetic tree and distribution of the AP2/ERF family in Solanaceae. (**A**) The maximum-likelihood gene tree was constructed for the AP2/ERF gene family, and the syntenic relationships between these genes were analyzed. Terminal branches represent nine categories, with each connecting line positioned within the circular gene tree indicating significant synteny conservation between gene pairs within subclades. (**B**) The copy numbers of various AP2/ERF categories across plant species. The number represented the level of enrichment among gene family members. The species names were displayed on the outer ring, accompanied by their corresponding abbreviations as documented in Supplementary Table S1. The identification of AP2/ERF categories was displayed on the inner ring of the figure. The various species and groups are visually distinguished by different colors.

To investigate the expansion process and genetic characteristics of the AP2/ERF gene family within the Solanaceae lineage, we conducted a targeted screening of AP2/ERF protein sequences. In this study, a total of 1282 AP2/ERF proteins were identified from 7 plant genomes, and these sequences were used to reconstruct a phylogenetic tree (Supplementary Table S5). Through an examination of the phylogenetic tree’s topology and branch support values, distinct groups within the AP2/ERF family were delineated. The bootstrap values associated with most main branches exceeded 85%, indicating a high level of stability and credibility of the classification results. The phylogenetic tree revealed the classification of AP2/ERF into three major classes and nine distinct subclades can be distinguished (Figure 1A). Different classes within the AP2/ERF family exhibit variation in their domain composition. Specifically, members of the AP2 subfamily contain two AP2 domains. The ERF-5 subfamily comprises proteins that have more than three AP2 domains, and other members of the AP2/ERF family typically include just one AP2 domain. An exception is that the members of the RAV subfamily possess B3 domains in addition to the AP2 domain. One group of seven AP2 homologs belongs to neither the AP2 nor the RAV clade and was termed “solo” for its ambiguity.

The AP2/ERF genes were conserved across all examined species, with a notably higher total number of copies observed in *Petunia axillaris* (Figure 1B and Supplementary Table S2). Each subfamily encompassed a comprehensive species composition, with sequence from all species in this study; however, the relative proportions varied. For example, ERF-1 exhibited a preference for *Solanum tuberosum*, while AP2 members were more prevalent in *Solanum pennellii*. Similarly, *Capsicum annuum* includes fewer RAV members but has a greater number of ERF-5 members. The AP2/ERF gene family in plants exhibits differential expansion across lineages, aligning with the birth–death model of gene family evolution, wherein genes generated through gene duplication can either be retained or lost over time [39].

The Solanaceae species possess multiple AP2/ERF copies, which are located on distinct chromosomes likely derived from a shared ancestral gene and subsequently underwent whole-genome duplication (WGD). We also found that the AP2/ERF tandem gene arrangement is widespread across Solanaceae (Supplementary Table S2). For example, there is one tandem gene cluster that includes five homologs in *Solanum tuberosum* and such tandem gene pairs in other species (Supplementary Table S2). The findings suggested that the amplification of AP2/ERF genes was driven not only by WGD but also by the presence of clusters of tandem repeat genes.

A noteworthy discovery is that the cross-clade tandem duplication of AP2/ERF genes was observed. Specifically, the gene pairs in *Solanum lycopersicum* (*sly_02g093130.1* and *sly_02g093150.2*), belonging to ERF-1 and AP2, respectively, exhibited a clear tandem sequential pattern in their arrangement (Figure 1A and Supplementary Table S3). This tandem arrangement, involving the ERF-1 and AP2 subfamilies, was also found in *Petunia axillaris*. The gene pairs *pax_00549g00114* and *pax_00549g10014*, despite their nomenclature implying distance, exhibit close proximity upon examination of their sequence locations, suggesting a concatenated duplication. Similar to the discovery of an ERF-1-AP2 tandem in *Solanum lycopersicum*, we also identified the same tandem duplication pairs (*Caba5523* and *Caba5525*; *Capana02g003060* and *Capana02g003062*; *spe_02g037700.1* and *spe_02g037720.1*; *stu_00004005* and *stu_00004006*) in the other species. This highlights that ERF-1 and AP2 genes originated from a tandem duplication event during the common ancestor of Solanaceae. The WGD event shared by Solanaceae further facilitated the amplification of the AP2/ERF gene family, ultimately resulting in its localization on distinct chromosomes.

### 2.2. Synteny Relations of the AP2/ERF Homologs

The synteny information of genomes, which refers to the similarity in gene arrangement on chromosomes across different genomes, can reflect the common origin of genomes and compare the rules of gene arrangement at a structural level (conserved or specific). The construction of an undirected synteny network related to AP2/ERF was undertaken in order to further investigate the conservation and divergence between members of the AP2/ERF and analyze the gene arrangement rule and species distribution within the cluster to extract the synteny characteristics of AP2/ERF in the genomes of Solanaceae species.

**Figure 2:**
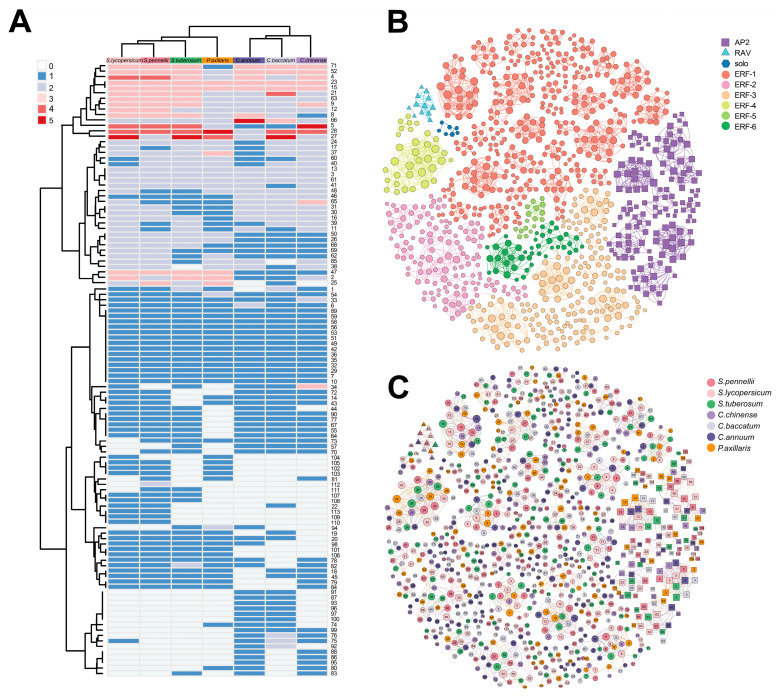
Phylogenetic profiling and synteny relationships of the AP2/ERF homologous genes in Solanaceae. (**A**) Phylogenomic profile of AP2/ERF syntelogs (syntenic homologous genes) across Solanaceae genomes. Species names are listed at the top of (**A**) as detailed in Supplementary Table S1. The corresponding phylogenetic tree of species is shown on the top side. Cluster IDs are indicated at the right of the figure. Columns represent syntelog clusters, and the color scale indicates the number of nodes (genes grouped in that cluster) per species. (**B**,**C**) Synteny network of the AP2/ERF gene family in Solanaceae species. Clusters from (**A**) can be divided into large-conserved clusters and lineage-specific clusters. The size of each node corresponds to the number of edges it has (node degree).

The network comprises 946 nodes, which represent the number of AP2/ERF genes located in the synteny regions, and 3021 edges that correspond to the number of paired synteny connections between these genes (Figure 2). When syntenic connections were plotted on the phylogenetic tree, the presence of robust synteny signals was evident within each branch (Figure 1A). This syntenic signal was in strong congruence with the observed phylogeny and supported the described gene phylogeny of the AP2/ERF subfamily in Solanaceae. It can be observed that not all AP2/ERF sequences present in the phylogenetic tree were reflected in the synteny network. For example, the tandem duplications of *Capsicum annuum* (*Capana01g004444*, *Capana01g004446*, *Capana01g004447*, and *Capana01g004449*) and *Petunia axillaris* (*pax_00078g00038*, *pax_00078g00042*, and *pax_00078g00043*) retained only one representative node each in the synteny network. This discrepancy may arise because certain genes are arranged in tandem, indicating their close proximity, and thus, only a representative member of the gene cluster is included in the synteny network to avoid redundancy.

On the network in Figure 2, most AP2/ERF genes form a distinct cluster by themselves, which is not connected to other subfamilies that have been identified. An exception is that syntenic connections were found between homologs of two subclade pairs: ERF-6 and ERF-3 (clusters 47 and 1). Similar patterns of shared synteny were detected for the ERF-2 and ERF-3 subclade pairs (clusters 75 and 64). The implication is that the functions of these AP2/ERF homologs may be analogous and facilitated by a shared genomic context. Furthermore, numerous instances have been observed where two nodes, located on distinct chromosomes within the same Solanaceae species and representing duplications of the AP2/ERF gene, were discovered to cluster together in a synteny cluster and demonstrate interconnectivity. The presence of this pattern confirms the occurrence of partial duplication of the AP2/ERF gene in the Solanaceae, resulting from WGD events. The conservation of synteny across different chromosomes provides compelling evidence for the impact of the WGD event on the distribution pattern of AP2/ERF genes in Solanaceae species.

### 2.3. Microsynteny Relations of the AP2/ERF Homologs

**Figure 3:**
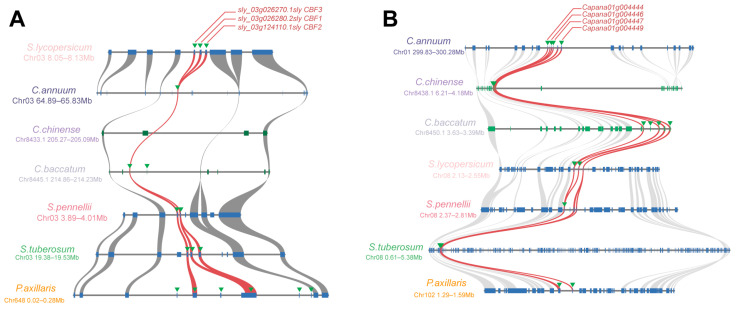
Microsynteny relationships of homologous genomic segments for two gene clusters in Solanaceae species. (**A**) Microsynteny relationships of conserved CBF homologous segments in Solanaceae species. (**B**) Microsynteny relationships of APE/ERF tandem gene clusters belonging to the ERF-1 subfamily in Solanaceae species. Syntenic relationships at the AP2/ERF loci are shown in Solanaceae species, including *Solanum lycopersicum*, *Capsicum annuum*, *Capsicum chinense*, *Capsicum baccatum*, *Solanum tuberosum*, *Petunia axillaris*, and *Ipomoea nil*. Gray curves connected the identified syntenic genes, while the annotated genes are represented by rectangles. Genes located on the forward strand are shown in blue and genes located on the reverse strand are shown in green. AP2/ERF genes were denoted by green arrows and connected by red lines.

The ERF subfamily, particularly ERF-1, constitutes a substantial proportion of the AP2/ERF family in Solanaceae, suggesting that it has undergone significant expansion during plant evolution. CBF, a member of the ERF-1 subfamily, is also known for its tandem duplication gene clusters. It plays a role in hypothermia response. Detailed microsynteny analysis of the CBF blocks revealed that the CBF sequences across Solanaceae exhibited a robust synteny relationship (Figure 3A). Not only does the flanking gene retain a high synteny relationship but the corresponding CBF homologous sequence is also shown in the synteny block of species in Solanaceae. We found varying numbers of CBF genes across the seven species studied. The highest number of CBF genes, totaling five, was identified in *Petunia axillaris*, all of which belong to the ERF-1 subfamily and tend to form tandem clusters. Solanum tuberosum contains the second most abundant number, with three CBF genes. It is worth noting that the copy number of the CBF gene in wild species was generally higher than that in cultivated varieties. The wild species *Solanum pennellii* manifested two copies of the CBF gene. The cultivated variety *Solanum lycopersicum*, which has undergone domestication, retains only a single copy of the CBF gene in contrast. It was similar that the *Capsicum baccatum*, *Capsicum annuum*, and *Capsicum chinense*, in which the degree of domestication was increasing, had 2, 1, and 0 CBF copies, respectively.

Similarly, we identified and analyzed another tandem gene cluster belonging to the ERF-1 subfamily. Considering the less stringent threshold for family screening, it is important to note that due to evolutionary processes, many genes with a common AP2/ERF ancestor have lost the AP2/ERF feature domain. This has led to a loss of identity among AP2/ERF family members. In the tandem gene cluster on Chromosome 2 (Figure 3B), the pepper species contained four AP2/ERF genes, while the tomato and petunia species had only one or two genes each, and the potato species had five AP2/ERF genes. While several genomic segments have undergone changes in terms of lineage, they still appear largely syntenic among Solanaceae.

### 2.4. Functional Diversification of AP2/ERF Revealed by Expression Profiles

**Figure 4:**
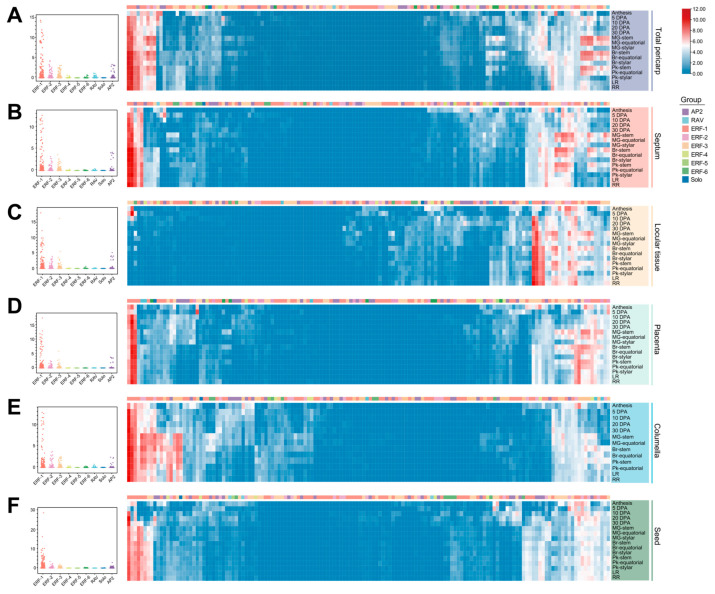
Expression patterns of AP2/ERF genes in *Solanum lycopersicum*. Expression profiles of candidate genes in tomato (M82) fruit are presented. (**A**) Expression patterns of AP2/ERF genes in tomato pericarp. (**B**) Expression patterns of AP2/ERF genes in tomato septum. (**C**) Expression patterns of AP2/ERF genes in tomato locular tissue. (**D**) Expression patterns of AP2/ERF genes in tomato placenta. (**E**) Expression patterns of AP2/ERF genes in tomato columella. (**F**) Expression patterns of AP2/ERF genes in tomato seed. The figure shows plots comparing expression levels among nine gene subfamilies, with the central line denoting the mean value. The data have been standardized. A color scale indicating expression levels is displayed on the right. Expression levels are normalized using a log2 scale. The differently colored top box denotes the diverse gene groups to which they belong. DPA, days post anthesis; MG, mature green; Br, breaker; Pk, pink; LR, light red; RR, red ripe.

**Figure 5:**
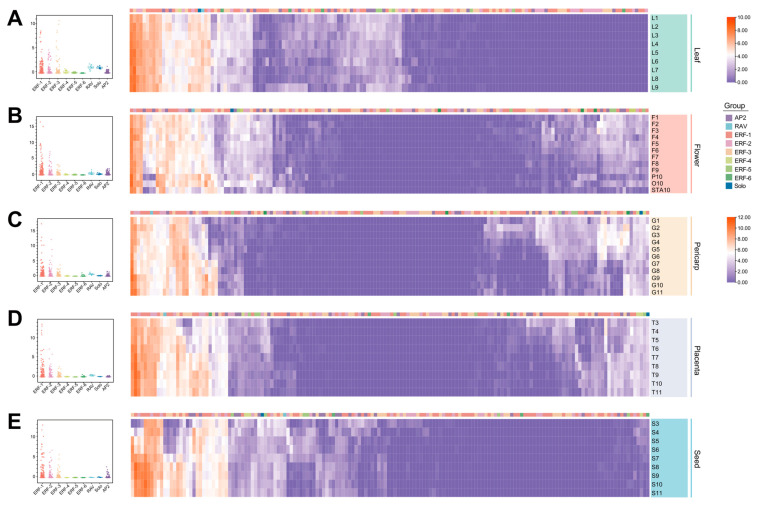
Expression patterns of AP2/ERF genes in *Capsicum annuum*. This figure illustrates the expression profiles of candidate genes within pepper tissues from the cultivar Zunla. Differences in expression among nine gene subfamilies are depicted, with the central horizontal line marking the mean expression level. The color scale representing the expression values for (**A**,**B**) is located at the top right. The color scale for (**C**–**E**) is positioned at the bottom right. The expression levels are normalized using log2.

To deepen our understanding of the retention and differentiation of expression patterns in Solanaceae, we conducted an analysis of AP2/ERF from the perspective of transcriptional regulation. We integrated transcript data from various tissues of *Solanum lycopersicum* (Figure 4) and *Capsicum annuum* (Figure 5) in developmental stages. The AP2/ERF genes were broadly expressed in organs but displayed both overlapping and differential expression patterns in various tissues of Solanaceae species. On the whole, the gene in the ERF subfamily exhibited homolog-expression bias, whereas the ERF-4/5/6 genes showed a distinct lack of transcriptional activity in development. In contrast, the genes in ERF-1/2/3 were generally highly expressed in all samples. Especially, the transcripts in parts of ERF-1 genes were abundant in both tomato and pepper organs, for example, *Capana03g002388* (pepper *CBF* gene), *Capana01g004446*, and *Capana01g004447*, suggesting that high expression genes may be essential as it serves as pivotal factors in plants. There was only one Solo protein identified in each genome, it exhibited low expression levels in both tomato and pepper tissues. The genes of the RAV subgroup exhibited consistently low expression levels in all detected tomato tissues (Figure 4). By contrast, the pepper AP2/ERF genes of subgroups RAV had high average expression preference in leaf, flower, pericarp, and placenta (Figure 4A), meanwhile subgroup solo showed high average expression levels in pepper leaf and flower (Figure 5A,B). It is speculated that gene function has further differentiated with the evolution of different species. Distinct patterns of gene expression across different tissues and developmental stages suggest that the multiple copies of AP2/ERF have obtained different functional evolution directions, catalyzing the diversification of roles among duplicated genes, which is of great significance for the adaptability of Solanaceae.

### 2.5. ERF-1 with Delayed Transition to Flowering

The ERF subfamily demonstrates a high copy number and complex synteny relationships, resulting from duplication events. These genes also play significant roles in transcriptional regulation within the genome (Figure 1 and Figure 2). The ERF-1 genes drew our attention because many members play diverse roles, such as involvement in floral development and flowering [40,41]. Tandem gene cluster 41 (Figure 3B and Supplementary Figure S1) contains four ERF-1 genes: *Capana01g004444*, *Capana01g004446*, *Capana01g004447*, and *Capana01g004449*. A multiple sequence alignment of the CDS revealed high homology within the gene cluster (Figure S2), particularly between genes *Capana01g004446* and *Capana01g004447* (91.30%). Additionally, the expression pattern of the *C. annuum CBF* gene *Capana03g002388* showed a strong correlation (*r*^2^) with those of *Capana01g004444*, *Capana01g004446*, and *Capana01g004447* (Figure S3). Based on this evidence, we predicted that *Capana01g004446* and *Capana01g004447* might share similar important biological functions with CBF. To investigate the function of these two highly homologous genes, we cloned them and generated gene-silencing pepper seedlings and gene-overexpression Arabidopsis plants, respectively.

**Figure 6:**
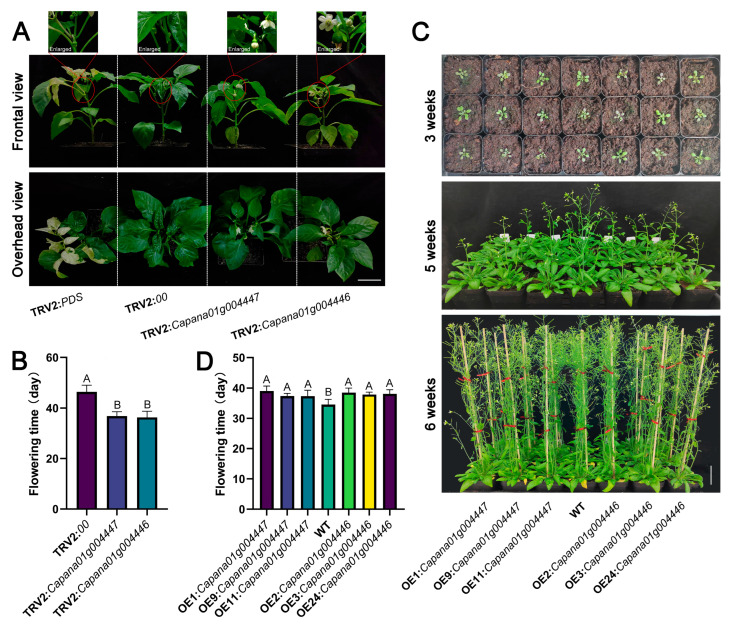
Phenotypes of flowering time in AP2/ERF-silenced pepper and AP2/ERF-overexpressed Arabidopsis. The phenotype and flowering time of gene-silenced plants are presented in (**A**,**B**), while those of gene-overexpressed Arabidopsis are shown in (**C**,**D**). Error bars indicate standard deviations (SDs) from three replicates. Data are presented as means ± SD. WT, wide type Arabidopsis; OE, Arabidopsis transgenic lines. Different uppercase letters indicate statistically significant differences from the WT or TRV2:00 group at *p* ≤ 0.0001, as determined by Tukey’s HSD. Scale bars = 2.5 cm.

Using multiple sequence alignments of genes CDS (Supplementary Figure S4) and SGN VIGS Tool, we constructed gene silencing plants in the pepper line R9. Photo-bleached leaves appeared in the TRV2:*CaPDS* pepper plants, serving as a positive control, approximately 40 days post-inoculation, indicating successful gene silencing. Compared to the TRV2:*00* controls, the expression of *Capana01g004446* and *Capana01g004447* was reduced by 84.25% and 74.18%, respectively, in various silencing pepper lines (Figure S5A). A visible phenotypic difference was observed between control and silenced pepper plants (Figure 6A). Flowering was delayed by approximately 10 days in pepper plants silenced for *Capana01g004446* or *Capana01g004447* compared to control plants (Figure 6B). To validate the results of VIGS, we successfully created Arabidopsis lines overexpressing *Capana01g004446* and *Capana01g004447*. We confirmed the DNA and mRNA levels of these genes using PCR and RT-PCR (Figure S5B). Similar to the VIGS results, overexpressing *Capana01g004446* or *Capana01g004447* in transgenic Arabidopsis caused a noticeable delay in flowering (Figure 6C). To ensure environmental factors did not influence the results, we repeated the observations under high-density planting conditions, and the overexpressed plants consistently exhibited delayed flowering (Figure S6). Collectively, these results indicate that the *Capsicum annuum* ERF-1 genes (*Capana01g004446* and *Capana01g004447*) exhibit a role in delaying the transition to flowering. This finding suggests that ERF-1 members might play a critical role in regulating the timing of floral initiation in *C. annuum*. Further investigation into the molecular mechanisms underlying this delay could provide insights into the regulation of flowering time in other Solanaceae species.

## 3. Discussion

Synteny, referring to the similarity in gene arrangement on chromosomes across different genomes, has proven to be an insightful approach to understanding the evolutionary connections among genes within and across species [42]. Combining synteny analysis with sequence similarity analysis is highly effective for inferring shared ancestry among genes and plays a crucial role in uncovering significant events associated with gene duplication and transposition [43]. Here, the AP2/ERF gene family was investigated for the first time in Solanaceae. We separated the AP2/ERF families and performed copy number and synteny analyses of individual subclades, comparing the relative conservation and specificity of synteny.

The AP2/ERF family has been classified into four major classes, with nine distinct subclades identifiable (Figure 1 and Figure 2). The AP2/ERF genes were found to be conserved across Solanaceae species. Phylogenetic tree and synteny analysis results revealed that the origin of AP2/ERF in Solanaceae can be attributed to an ancestral tandem duplication event. This tandem duplication likely served as the genetic foundation for the diversification of AP2/ERF within the family. The subsequent expansion of AP2/ERF genes in the Solanaceae appeared to have been primarily driven by WGD events within this lineage. Such genomic events are known to contribute to functional redundancy and the evolution of new traits, thereby enhancing metabolic flexibility and adaptation to varying environmental conditions [44]. Additionally, there were variations in the domain composition among different classes within the AP2/ERF family. Consistent with previous studies, proteins belonging to the AP2 subfamily possess two highly similar and serially repeated AP2 domains, while RAV-like AP2/ERF transcription factors contain one AP2 domain and one B3 domain [45,46]. Notably, we identified a distinct clade (ERF-5) within the ERF subfamily that contains more than three AP2 domains, contrasting with the typical ERF subclades that have only one AP2 domain. The potential functional implications of these additional AP2 domains warrant further investigation.

The ERF subfamily, constituting a significant portion of the AP2/ERF family in Solanaceae, has undergone substantial expansion and gene retention throughout plant evolution. Our data show that the majority of ERF proteins possess a highly conserved AP2 domain and belong to a large multigene subfamily of plant-specific transcription factors, comprising over 100 members (Figure 1B). Furthermore, the ERF subfamily can be subdivided into six distinct subclades. Specifically, the identification of syntenic connections between homologs of two subclade pairs, ERF-6 and ERF-3 (clusters 47 and 1), as well as ERF-2 and ERF-3 (clusters 75 and 64), provides further evidence of gene duplication and divergence within the ERF gene family in Solanaceae (Figure 2). Our study focused on ERF-1 because it includes the “star gene” CBF, known for its tandem duplication gene clusters. Gene duplication retention is a crucial mechanism driving the evolution of genes and their associated functions [47]. While some duplicate copies resulting from gene duplication are quickly lost through pseudogenization, a certain proportion remains conserved. These “backup” genes can undergo rapid evolution and become essential in fundamental biological processes [48]. Tandem duplication, in particular, generates a large number of gene copies and allelic variations within a population, playing a vital role in enabling plants to adapt to rapidly changing environments [49]. We observed varying numbers of CBF genes across the seven Solanaceae species studied. Notably, the copy number of the CBF gene in wild species was generally higher than in cultivated varieties. Overexpression of CBFs has been shown to enhance cold tolerance in transgenic species, and CBFs have been identified as a major quantitative trait locus for freezing tolerance [10,11]. This suggests that wild relatives may have retained greater genetic diversity, contributing to their enhanced adaptability to low-temperature conditions.

Similarly, we identified and analyzed another tandem gene cluster within the ERF-1 subfamily. There are slight differences in the number of recognized ERF sequences among different species. Microsynteny analysis revealed that some sequences, while showing collinearity with ERF genes, are not recognized as ERF. This may indicate that, over the course of evolution, the integrity of the characteristic domains of these sequences has been compromised, leading to the loss of their ERF identity.

The extensive AP2/ERF family is involved in numerous physiological processes in plants [7,9,10,12,50]. The study of AP2/ERF CBF genes is a current research hotspot. The research progress of the CBF gene cluster (C-repeat binding factors, also known as DREB1 gene cluster) mainly focuses on the response mechanism of plants to abiotic stress, which activates the expression of a series of downstream cold response genes to help plants cope with cold, drought, and other abiotic stresses [8,12,51]. *Capsicum frutescens CfCBF3* (HM748942) was cloned and overexpression of *CfCBF3* under the control of the CaMV35S promoter in tobacco increased chilling tolerance without a dwarf phenotype [51]. In our study, the abundance of CBF genes in the Solanaceae family varied significantly (Figure 3A), suggesting that different genera within the Solanaceae family may have varying degrees of resistance to low temperatures.

AP2/ERF genes exhibited differential expression across various tissues (Figure 4 and Figure 5). For example, many AP2/ERF genes showed higher expression levels in roots, a finding consistent with previous research in Chinese cabbage and *Brachypodium distachyon* [35,41,52]. These genes possess specific biological functions. The rice *OsERF71* and *OsERF48* have been shown to modify rice root architecture and improve drought resistance [14,53]. Tomato CaAP2/ERF genes showed increased expression during tomato fruit ripening (Figure 4). Similarly, studies in tomatoes have indicated that ERFs play a role in regulating fruit ripening [54]. Furthermore, the Arabidopsis *CBF1*, *CBF2*, and *CBF3* genes have been identified, and the constitutive overexpression of these three genes leads to late flowering [50]. In this study, pepper ERF-1 genes were highly expressed in pepper flowers, including the pepper CBF genes *Capana03g002388*, *Capana01g004446*, and *Capana01g004447* (Figure 5). Functional verification experiments also demonstrated that *Capana01g004446* and *Capana01g004447* play a role in delaying the transition to flowering (Figure 6). The findings regarding the AP2/ERF transcription factor homolog CaAP2/CaFFN also support our results [24,41]. We employed two functional verification approaches: the Capsicum VIGS assay and overexpression in Arabidopsis. The Capsicum VIGS assay is an instantaneous silencing technique that can be influenced by the silencing efficiency, which may vary depending on the specific gene and environmental conditions. In contrast, overexpression in Arabidopsis represents a heterologous expression system, which may not fully replicate the native context of the target gene. Both of these methods, while valuable, have certain limitations and may deviate to some extent from the true function of the target gene. These potential deviations should be considered when interpreting the results, and further studies using complementary approaches, such as stable transformation in the native species, may be necessary to confirm the findings. In summary, our study employed a phylogenomic approach, combined with a meticulous analysis of synteny relationships, to shed light on the early origin and evolutionary trajectory of AP2/ERF genes in Solanaceae plants. A deeper understanding of the evolutionary history of these genes enhances our insights into the mechanisms that shape their functions.

## 4. Materials and Methods

### 4.1. Collection of Solanaceae Genomes

We collected seven Solanaceae genomes from comprehensive databases for this study, including *Solanum pennellii*, *Solanum lycopersicum*, *Solanum tuberosum*, *Capsicum chinense*, *Capsicum baccatum*, *Capsicum annuum*, and *Petunia axillaris*. Detailed information about the genomes used in this study can be found in Supplementary Table S1. Each genome underwent a standardized naming preprocessing step, and only the longest transcript was retained, along with the corresponding BED/GFF annotation files, for subsequent analyses.

### 4.2. The Identification of AP2/ERF Members in Solanaceae Plants

The conserved domain (PF00847) of AP2/ERF proteins, which is part of the AP2/ERF superfamily, was obtained from the Pfam database (http://pfam.xfam.org/) (accessed on 27 September 2022), and a Hidden Markov Model (HMM) was constructed. Using HMMER3 [55], an exhaustive search was conducted to identify all AP2/ERF sequences with an E-value threshold of 0.001 from the Solanaceae genomes of seven representative species. Sequences were then annotated, categorized, and extracted by referencing the previously reported AP2/ERF sequences [30].

### 4.3. Sequence Alignment and Phylogenetic Analysis

The protein sequences were aligned using the L-INS-I strategy in MAFFT [56]. Poorly aligned positions and spurious sequences were removed using trimAl v1.2rev59 [57]. Maximum-likelihood trees were constructed using Iq-Tree v2.1.2, employing the best-fit model Q. plant + G4 with 1000 bootstrap replicates [58,59]. Phylogenetic trees were visualized and annotated using iTOL (http://itol.embl.de) (accessed on 19 October 2022).

### 4.4. Detection of Syntenic Blocks and Construction of Synteny Network

Collinearity refers to the conservation of gene sequences on chromosomes through evolution, reflecting significant relationships between genomic contexts in terms of gene function and regulation. This allows for the examination of both large-scale (e.g., genome rearrangements and duplications) and small-scale (e.g., gene translocations, insertions, and deletions) molecular evolutionary events across species. The collinear network analysis method developed in our laboratory effectively addresses the challenges posed by pairwise species comparisons. It transforms traditional pairwise or horizontal comparisons into the analysis of gene cluster networks, providing a powerful tool for organizing and utilizing collinear data from multiple sequenced species. This approach enables the identification of previously undiscovered complex evolutionary relationships within various gene families [60].

The computation of synteny blocks and the construction of the synteny network were carried out using the SynNet-pipeline synteny analysis developed by Zhao and Schran (https://github.com/zhaotao1987/SynNet-Pipeline) (accessed on 6 October 2022). Protein sequence similarity searches were conducted using Diamond v0.9.1 [60]. MCScanX [61] with default parameters was used to detect all possible syntenic associations between and within the genomes of different species. Syntenic blocks related to the AP2/ERF gene were extracted from the comprehensive synteny database to construct the target synteny network. Phylogenomic profiles were produced by counting the number of syntenic genes in each genome for each synteny cluster. The synteny relationships were annotated on the phylogenetic tree using iTOL, and the constructed network was visualized and annotated using Gephi v0.9.1 [62].

### 4.5. Microsynteny Analysis of AP2/ERF and Flanking Genes

We conducted microsynteny analysis on AP2/ERF gene-centric blocks, which included the AP2/ERF genes as well as their upstream and downstream flanking genes. The pairwise synteny regions were identified by MCScanX (Python version). Microsynteny analysis and visualization were generated using JCVI (https://github.com/tanghaibao/jcvi/wiki/Mcscan-(python-version)) (accessed on 15 November 2022).

### 4.6. The Analysis of Genes Expression Pattern

For tomatoes, gene expression in various organs and at different fruit developmental stages was analyzed in Solanum lycopersicum M82 from NCBI accession SRP109982 [43]. Transcriptome data for pepper genes from the Zunla-1 genome were obtained through the Pepper Hub (http://lifenglab.hzau.edu.cn/PepperHub/index.php) (accessed on 5 January 2022) [63].

### 4.7. Cloning and Vector Construction of Functional Genes

Full-length sequences of functional genes were amplified from cDNA extracted from the leaves of the pepper line R9 (obtained from the World-Asia vegetable research and development center, PP0042-51), using the pVBG2307-*gene*-OE primers (Supplementary Table S4). The amplified fragments were cloned into pMD19-T (TaKaRa, Dalian, China) and subsequently subjected to sequencing analysis.

To evaluate the functions of genes in pepper, the tobacco rattle virus-based VIGS method was used to silence the target genes. A specific 132 bp fragment (for gene *Capana01g004446*) or 135 bp fragment (for gene *Capana01g004447*) was amplified from the pepper line R9 using TRV2-*gene*-VIGS primers designed with the SGN VIGS Tool (https://vigs.solgenomics.net/) (accessed on 10 February 2022) (Supplementary Table S4). The PCR products were then inserted into the TRV2 vector to create the respective TRV2:*gene* constructs.

Overexpression vectors containing the CaMV35S promoter were constructed by combining them with the functional gene sequences amplified from the R9 cDNA using the pVBG2307-*gene*-OE primers (Supplementary Table S4).

### 4.8. Phenotypic Characterization of Transgenic and Gene-Silenced Plants

We used a breeding pepper line (R9) and wild-type (WT) *Arabidopsis thaliana* plants (ecotype Columbia) for gene silencing and overexpression experiments, respectively. Seedlings were cultivated in a climate-controlled environment with a 16 h light/8 h dark cycle and 60 to 70% relative humidity. The temperatures were maintained at 25 °C during the day and 18 °C at night for pepper and 22 °C during the day and 18 °C at night for Arabidopsis. Silenced pepper plants were produced using the method detailed in our previous study [64,65]. The blank vector TRV2:*00* served as the control, and TRV2:*CaPDS* (encoding phytoene desaturase) was used as a positive control for successful gene silencing. Subsequently, qRT-PCR analysis [65] was performed to measure the silencing efficiency of the target genes compared to the control.

Gene overexpression was conducted according to methods described previously [65,66]. Positive transgenic lines were confirmed through PCR and RT-PCR using DNA and RNA detection primers (Supplementary Table S4). The T3 generation seeds of the transgenic lines were used for the experimental analysis.

The flowering time in pepper and Arabidopsis can be accurately defined by recording the time of the initial flower openings. This temporal measurement was initiated from the point of seed imbibition in the soil.

## Data Availability

All datasets generated for this study are included in the manuscript and Supplementary Material.

## Supplementary Materials

The following supporting information can be downloaded at https://www.mdpi.com/article/10.3390/ijms252011247/s1.
