# Supplementary material for: Phylogenomic Analysis and Functional Characterization of the APETALA2/Ethylene-Responsive Factor Transcription Factor Across Solanaceae
Source: Int J Mol Sci. 2024 Oct 19;25(20):11247. doi: 10.3390/ijms252011247 (PMC11508751; doi:10.3390/ijms252011247)
Supplement: Supplementary file 1 [file ijms-25-11247-s001.zip › Supplementary Figure.pdf]

Supplementary Material

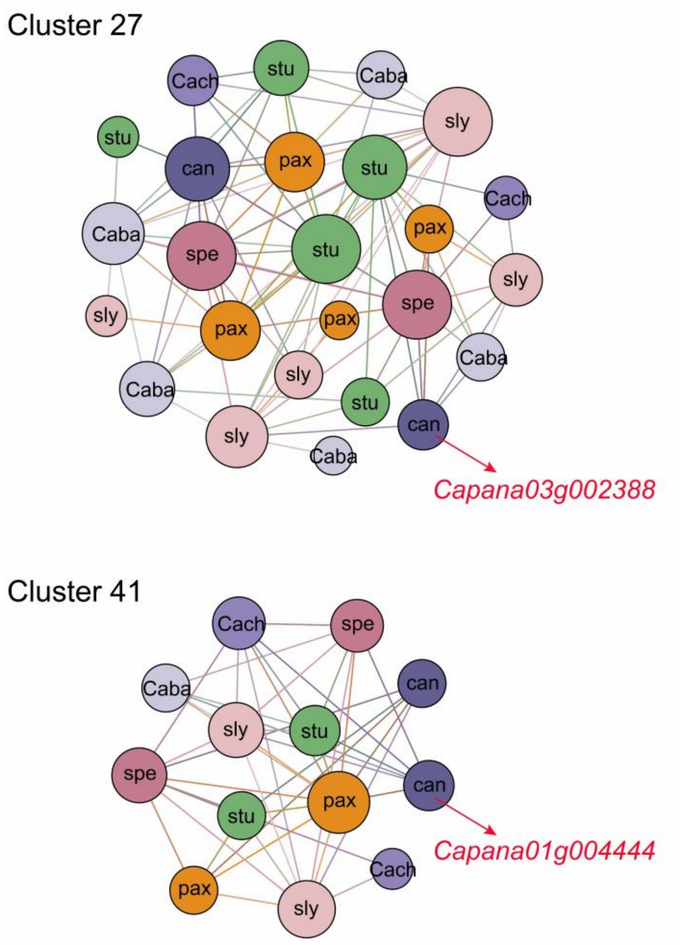

Figure S1. Synteny network of the clusters 27 and 41 in Solanaceae species. The pepper genes *Capana03g002388* and *Capana01g004444* are indicated.

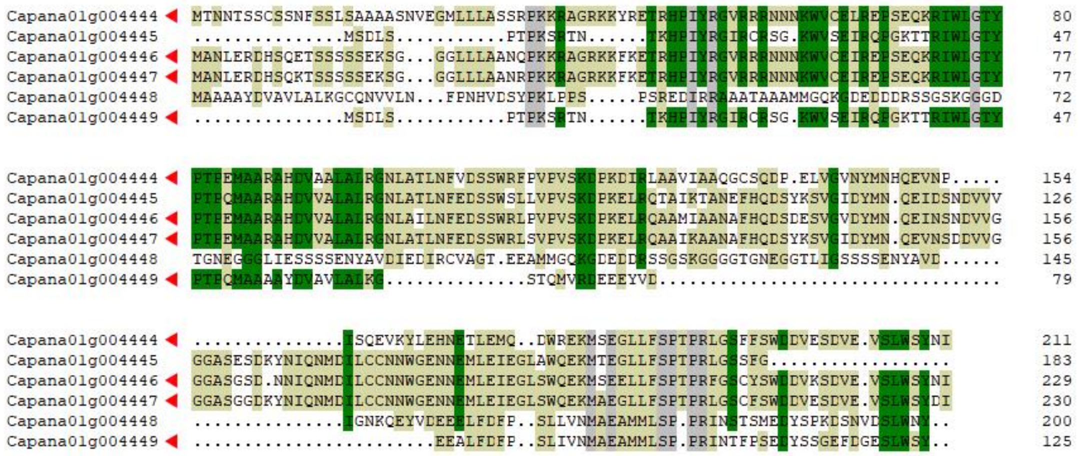

Figure S2. Multiple sequence alignment of pepper proteins. Clustal Omega software (http://www.clustal.org/) was used to align the protein sequences using default parameters and the results were minor repaired by DNAMAN Version 9.0 (www.lynnon.com) software.

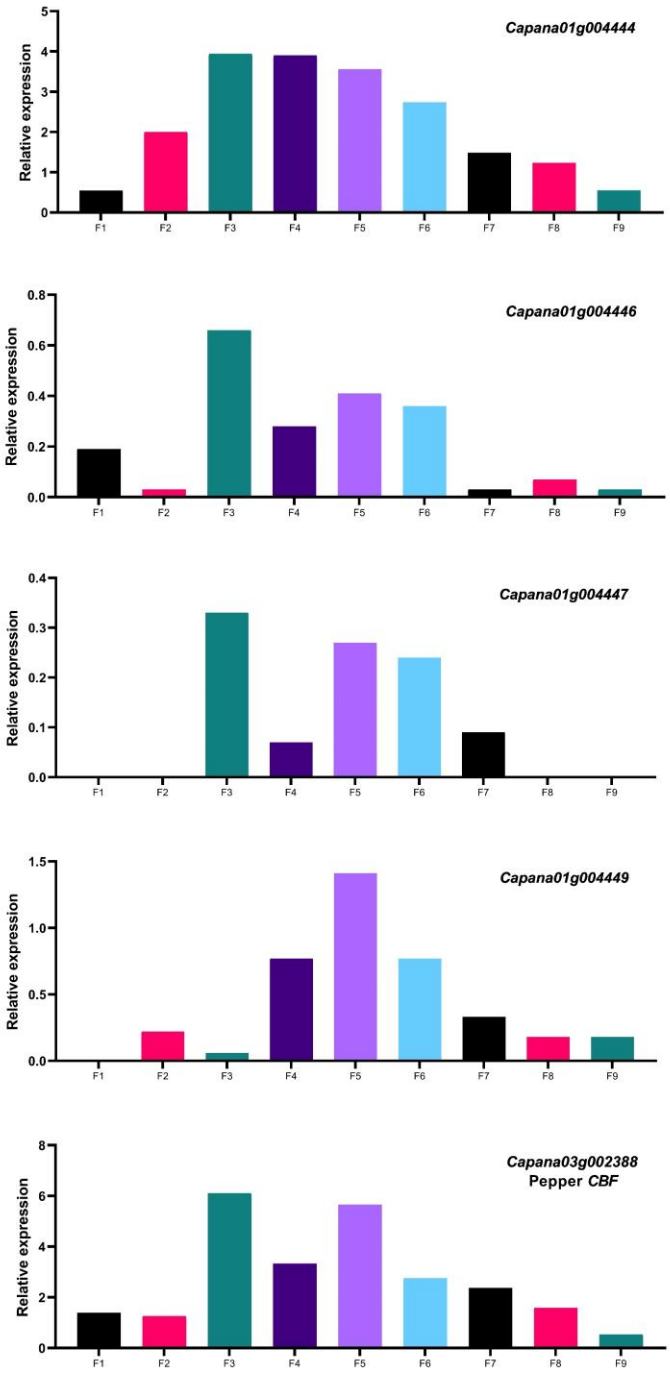

	Capana03g002388 vs. Capana01g004444	Capana03g002388 vs. Capana01g004446	Capana03g002388 vs. Capana01g004447	Capana03g002388 vs. Capana01g004449
Pearson r^2	0.7373	0.7957	0.8135	0.2485
P	**	**	***	ns

Figure S3. Expression of pepper ERF/AP2 genes during pepper flower development. The expression correlation between *Capana03g002388* and the cluster genes (*Capana01g004444*, *Capana01g004446*, *Capana01g004447*, and *Capana01g004448*) during the development of pepper flowers. Pearson's correlation coefficients r^2 are provided.

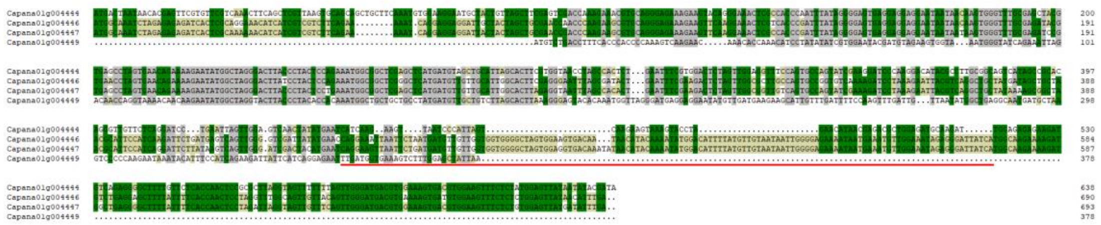

Figure S4. Multiple sequence alignment of pepper gene CDS. Clustal Omega software (http://www.clustal.org/) was used to align the DNA sequences using default parameters and the results were minor repaired by DNAMAN Version 9.0 (www.lynnon.com) software.

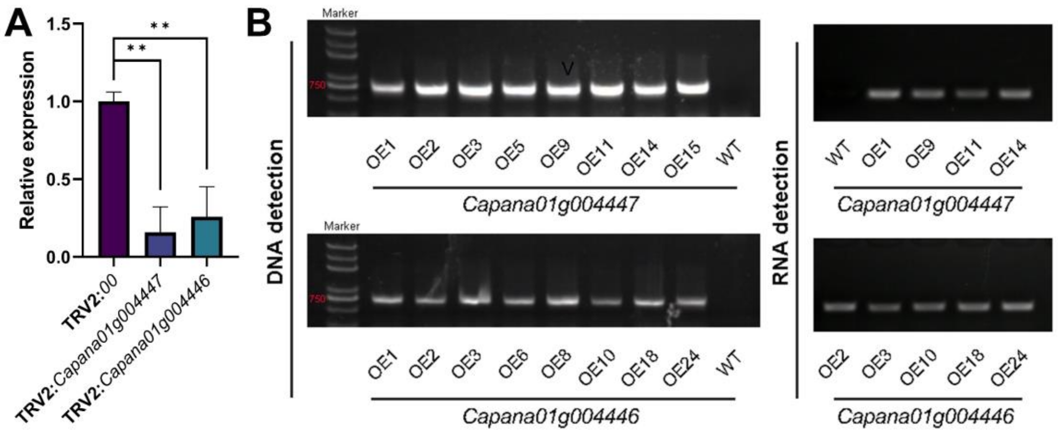

Figure S5. Confirmation of the *gene*-silenced pepper and *gene*-overexpressing Arabidopsis. (A) The efficiency of gene expression silencing in *CaHsfA1d*-silenced peppers. The primers qPCR-*Capana01g004446*/*Capana01g004447* were used to detect the expression of *Capana01g004446*/*Capana01g004447*. The *CaUBI3* gene was used as a reference with qPCR-*CaUBI3* primers. ** indicates significant difference at the 0.01 levels by *t*-test. **(B)** PCR was performed using the primers Detection-Capana01g004446 and Detection-Capana01g004447 to detect the target genes at both the DNA and RNA levels. WT, wide type *Arabidopsis*; OE, Arabidopsis transgenic lines.

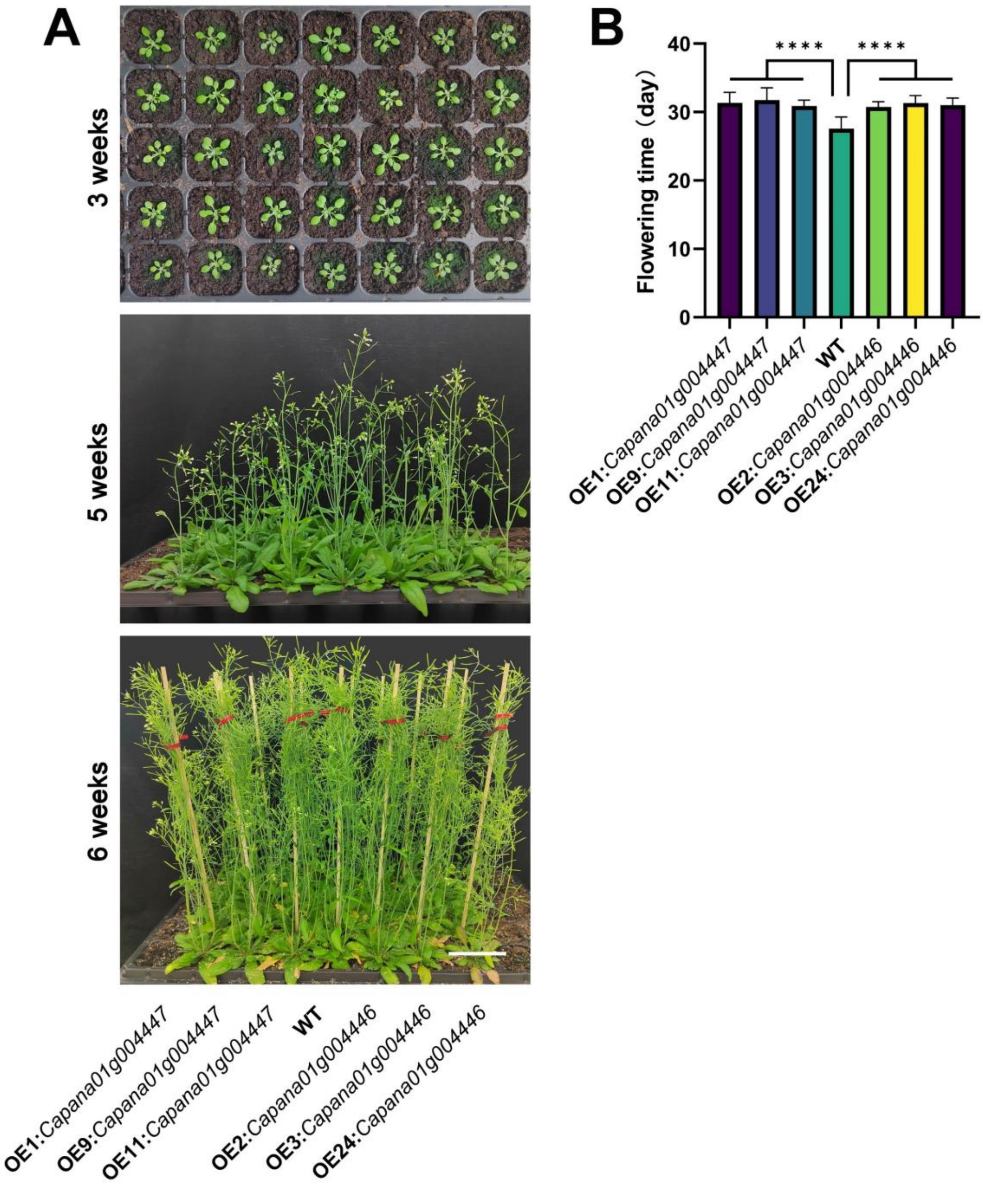

Figure S6. Phenotypes of flowering time in AP2/ERF-overexpressed Arabidopsis. The phenotype of gene-overexpressed Arabidopsis is presented in Figures S6A, while the flowering time is shown in Figures 6B. Error bars indicate standard deviations (SDs) from three replicates. Data are presented as means ± SD. Significance was determined by Student's *t*-test (****P < 0.0001). Scale bars = 5 cm.

Table S4 Primers used in this study

Primer name	Primer Sequence (5'→3')
**For vector construction**	
pVBG2307-*Capana01g004446*-OE	F: CGCGGATCCATGGCAAATCTAGAGAGAG<br>R: CCGGAATTCTCAAATGTTATAACTCCAGA
pVBG2307-*Capana01g004447*-OE	F: CGCGGATCCATGGCAAATCTAGAGAGAGATC<br>R: CCGGAATTCTCAAATATCATAACTCCACAGAG
TRV2-*Capana01g004446*-VIGS	F: CCGGAATTCCAGGAAATTAATTCTAATGATGTTGT<br>R: CCGCTCGAGTGATAATCCCTCTATTTCCAACAT
TRV2-*Capana01g004447*-VIGS	F: CCGGAATTCCAGGAAGTTAATTCTGATGATGTTG<br>R: CCGCTCGAGTGATAATCCCTCTATTTCCAACAT
**For DNA/RNA detection**	
Detection-*Capana01g004446*	F: ATGGCAAATCTAGAGAGAG<br>R: TCAAATGTTATAACTCCAGA
Detection-*Capana01g004447*	F: ATGGCAAATCTAGAGAGAGATC<br>R: TCAAATATCATAACTCCACAGAG
qPCR-*CaUBI3*	F: TGTCCATCTGCTCTCTGTTG<br>R: CACCCCAAGCACAATAAGAC
qPCR-*Capana01g004446*/*Capana01g004447*	F: ATCGTCGTCTTCAGAAAAATCAG<br>R: CGAGTTTCCTTGAACTTCTTTCT
